# Matching Patient and Therapist Anaclitic–Introjective Personality Configurations Matters for Psychotherapy Outcomes

**DOI:** 10.1007/s10879-018-9389-8

**Published:** 2018-03-14

**Authors:** Andrzej Werbart, Mikael Hägertz, Nadja Borg Ölander

**Affiliations:** Department of Psychology, Stockholm University, 106 91 Stockholm, Sweden

**Keywords:** Patient–therapist match, Personality configurations, Outcomes, Psychoanalytic psychotherapy, Young adults

## Abstract

Decades of psychotherapy research suggest that patient–therapist match accounts for outcome beyond single patient or therapist variables. This study examines the associations between different patterns of patient–therapist matching (in terms of orientation on relatedness or self-definition) and outcomes at termination of psychoanalytic psychotherapy with young adults. Thirty-three patients and their therapists were classified as predominately anaclitic or introjective at baseline. Patients in the convergent patient–therapist dyads (both anaclitic or both introjective) showed significantly greater symptom reduction and increased developmental levels of representations of mother than patients in the complementary dyads (opposite personality configurations). Moreover, convergent patient–therapist match was connected with larger effect sizes on all outcome measures and lower proportion of non-improved patients. These findings suggest the importance of the therapists’ early adjusting their orientation on relatedness or self-definition to their patients’ predominant personality configuration in order to enhance treatment outcomes.

## Background and Aims

The crucial role of the therapeutic relationship in the change process and outcome, as well as the therapist’s significant contribution to that relationship (Del Re et al. 2012), is increasingly acknowledged in psychotherapy research (Norcross and Wampold 2011a). Different patients require not only different treatments but also different therapeutic relationships (Norcross and Wampold 2011b). The personality of the therapist does influence the psychotherapeutic process. A recent systematic literature review (Lingiardi et al. 2018) confirmed what every clinician knows: the therapist’s subjective characteristics influence results of psychodynamic psychotherapists. According to this review, therapists’ interpersonal functioning, reflective and introspective capacities, and specific personality characteristics showed the strongest evidence of a direct effect on treatment outcomes. It can be assumed that these therapists’ variables were complementary (opposite) to the patients’ inabilities.

Decades of psychotherapy research suggest that patient–therapist match accounts for outcome beyond single patient or therapist variables. This issue touches upon the challenging question of the nature of matching. Accordingly, matching what does matter? Matching more objective or more subjective characteristics? Convergent match based on similarities or complementary match based on opposites? The research findings are inconsistent.

Most studies of patient–therapist match try to demonstrate that similar sex, socioeconomic status, ethnicity, or more generally, a shared cultural background with similar attitudes, values, and believes influence the early therapeutic relationship, session content, continuation rates, treatment satisfaction, and outcomes (e.g., Cabral and Smith 2011; Ibaraki and Hall 2014; Reis and Brown 1999). However, contrasting convergent and complementary patient–therapist match might be too simplistic. As early concluded by Beutler et al. (1991), optimal pairings comprise therapists who share similar humanitarian and intellectual values with their patients, but have discrepant views of personal safety and the value of interpersonal intimacy and attachment.

Attachment to the therapist has been conceptualized as an important ingredient in the therapeutic relationship (Mallinckrodt 2010). Furthermore, the therapist’s attachment style was demonstrated to affect the patient and the therapeutic relationship (Slade 2016). Following this line of research, several studies focused on matching the patient’s and the therapist’s attachment styles, most of them supporting the complementarity hypothesis (e.g., Bruck et al. 2006; Petrowski et al. 2011). Other studies concluded that convergent attachment patterns further good outcomes for severely disturbed or high-avoidant patients (Farber and Metzger 2009; Wiseman and Tishby 2014).

The complementarity hypothesis was also supported by early studies of the patient–therapist match on personality variables, assessed using the MMPI and the Omnibus Personality Inventory (Dougherty 1976), applying the type A and type B dichotomy (Anderson and Carter 1982), or the Check List of Interpersonal Transactions (Kiesler and Watkins 1989). Other studies supported the similarity hypothesis, such as matching cognitive structures (measured by the Interpersonal Discrimination Test; Hunt et al. 1985), similarity on the Five-Factor Personality Inventory (Coleman 2006), or fitting personality types (assessed with Self-Directed Search; Taber et al. 2011).

Despite different conceptualizations and operationalizations, the studies of personality match hint at the dimensions of mode of relating/affiliation and autonomy/self-boundaries. These are also the polarities of experience, central to Blatt’s (2008) empirically anchored theory of personality development, psychopathology, and the therapeutic process. According to his double helix model, the main mechanism of change in psychoanalytic therapy is reactivation of normal developmental processes and new internalizations in the context of the therapeutic relationship (Blatt et al. 2008; Luyten and Blatt 2013). This involves alternating sequences of *gratifying involvement* (attachment) and *experienced incompatibilities* (separation, or some disruption to a gratifying relationship), leading to more mature forms of relatedness and a more integrated sense of self (Behrends and Blatt 1985; Blatt and Behrends 1987). Development of the sense of self (the introjective line) leads to increasingly mature levels of interpersonal relatedness (the anaclitic line) that, in turn, facilitates further differentiation and integration in the development of the self (Blatt and Luyten 2009; Luyten et al. 2013). Psychological health involves both a meaningful identity and meaningful attachments, i.e. a balance between differentiation and relatedness, autonomy and intimacy. Still, most individuals, also within the normal range of psychological development, have an inclination towards either the relatedness dimension or the self-definition dimension. In contrast, different forms of psychopathology reflect an exaggerated and distorted preoccupation with one or the other of these developmental dimensions (Luyten and Blatt 2013). The anaclitic configuration is connected with difficulties in close relationships and attachment anxiety, while the introjective configuration is connected with excessive demands for achievement and perfectionism, and with attachment avoidance (Luyten and Blatt 2013). Disturbances in each developmental line may lead to the same symptoms, but require different treatments. Introjective depression, based on the sense that “I am a failure,” responds to classical psychoanalysis, with the therapist as a listener, helping to elicit growth in an independent sense of self. Anaclitic depression, based on the feeling that “I am not worthy of love,” is effectively treated by a more assertive therapist, guiding the formation of relationships (Blatt et al. 2010). Nonetheless, following Blatt’s model, the goals of psychoanalytic psychotherapy include enhancing the patient’s capacities for both being together and autonomy, both relationships and self-other differentiation.

However, this may be not only a matter of therapeutic technique, but also of the therapist’s personality orientation. Heinonen and Orlinsky (2013) studied the interplay between therapists, personal identities, theoretical orientations, and professional relationships. They concluded that the therapist’s professional self, underlying interactions with patients, is rooted in the general self-experience in close personal relationships. However, it is possible that different aspects of the therapist’s personality are actualized with different patients. Accordingly, we still need studies focusing on the therapists’ personality configuration as actualized in relationship to their specific patients.

Based on these issues, the objective of the present study is to examine how the therapist’s personality configuration (anaclitic orientation on relatedness/affiliation or introjective orientation on self-definition/autonomy) is actualized, manifests itself and interacts with the patient’s personality configuration early in the therapeutic relationship. Furthermore, we examine the associations between different patterns of patient–therapist matching (convergent or complementary personality configurations) and outcomes at termination of psychotherapy with young adults. How are the therapeutic dyads distributed at baseline between different matching patterns? Do the different groups of matching patterns show different outcomes at termination (in terms of symptom reduction and of changes in the inner representational world)? Following previous studies (Werbart et al. 2017), we expected more pronounced improvements in the complementary groups.

## Methods

### Setting

The present study is based on archival data from the *Young Adult Psychotherapy Project* (YAPP), a longitudinal, prospective, naturalistic study of psychoanalytic psychotherapy at the former Institute of Psychotherapy in Stockholm, Sweden. The project was approved by the Regional Research Ethics Committee at the Karolinska Institutet and all participants gave their informed consent. Of the total of 134 patients (vast majority of them self-referred) aged 18–25, 92 applied for individual psychotherapy. The main complaints, presented in pretreatment interviews, were low self-esteem (97%), depressed mood (66%), anxiety (55%), and conflicts in close relationships (66%) (Wiman and Werbart 2002).

The psychotherapies were conducted in accordance with standard descriptions and procedures of psychoanalytic psychotherapy. The treatments generally aimed at helping the young adults to overcome developmental arrest and to better handle strains in everyday life. The goals, duration and frequency of therapy were adjusted to individual patient needs and formalized in a written, renegotiable contract between therapist and patient. The treatments were conducted by 37 therapists who all shared a psychoanalytical frame of reference, even though working quite autonomously with varying preferences regarding theory and technique. No manual was used, and treatment fidelity was not controlled for. However, the therapists met weekly in clinical teams to discuss clinical experiences and treatment problems. All included treatments ended by mutual agreement. The patients in individual psychotherapy (with the nine nonstarters excluded) stayed in treatment for a mean of 24.6 months (*SD* = 16.3; *Mdn* = 21; range 2–85) with a frequency of one or two sessions per week. All outcome measures except the developmental level of the representation of mother changed significantly from pretreatment to termination, and all outcome measures showed significant improvements from pretreatment to the 1.5-year follow-up (Lindgren et al. 2010).

### Participants

To be included in the present study, patient and therapist interviews as well as outcome data pretreatment and at termination had to be available. Owing to the research design, the participants were interviewed at baseline in only every second case. Thus, of the 92 therapeutic dyads in individual therapy, 33 could be included. The patient data included in the present study were derived from a previous investigation of changes in the patients’ anaclitic–introjective personality configurations following psychotherapy (Werbart et al. 2017).

Twenty-seven of the patients were women and six were men. The average age at the start of psychotherapy was 22.3 years (*SD* = 2.1; range 18–25). Fourteen patients lived alone, 10 lived with a partner, seven lived with their parents, and two lived with a friend. None were married or had a child. The most common occupation was full-time study (20 patients) followed by full-time work (eight patients) and work in combination with studies (four patients); one patient was on sick leave. Thirty-one patients were born in Sweden; five had at least one parent of foreign origin. In all, 24 patients had at least one parent with a university degree, thus indicating a high socioeconomic status within their family of origin.

Psychiatric diagnoses in accordance with DSM-IV-TR (American Psychiatric Association 2000) Axis I were made retrospectively by two independent experts based on interview transcripts, case-book notes, and other available research and clinical data. The interrater agreement was tested based on the assessment of 20 cases, and was found to be satisfactory (Cohen’s kappa κ = 0.71). Consensus diagnoses were used in further analyses. Personality disorders were diagnosed by the patients’ therapists by completing checklists covering all the general and specific criteria of Axis II personality disorders. Twenty-three patients had one or more Axis I-diagnoses: six had anxiety disorder, 10 had depression, two had obsessive–compulsive disorder, two had alcohol or substance abuse, and one had acute stress disorder. Twelve patients fulfilled criteria for one or more personality disorders (PD): in cluster A, two had paranoid PD; in cluster B, two had borderline PD and two had antisocial PD; in cluster C, three had avoidant PD. Furthermore, three patients had personality disorder not otherwise specified, three had the research diagnosis of depressive PD, and one had passive-aggressive PD; four patients had no psychiatric diagnosis. Fifteen patients had previous outpatient or inpatient psychiatric contact; 13 had previous psychotherapeutic contact.

The mean treatment duration was 23.7 months (range 7–55; *SD* = 12.6). At baseline, the 33 patients were treated by 21 therapists; 14 female and seven male. Their mean age at the start of treatments was 56 years (range 36–64; *Mdn* = 58; *M* = 56.2; *SD* = 6.8). Ten therapists were social workers, nine were psychologists and two were psychiatrists. All but one of the therapists were licensed psychotherapists with two to 15 years’ experience after being licensed (*Mdn* = 13, *M* = 10.6, *SD* = 4.2), seven of them being psychoanalysts, and each working as a teacher and supervisor in an advanced psychotherapy training program. One therapist had basic psychotherapy training. Two therapists had three patients, eight had two patients, and 11 had one patient each. As the analysis unit in the present study is the patient–therapist dyad, each therapist in the 33 treatments is assessed and included in the data analysis as a unique entity.

### Interviews

The participants were interviewed prior to psychotherapy and at termination using the Object Relations Inventory (ORI; Diamond et al. 1990; Gruen and Blatt 1990; for review see; Huprich et al. 2016). The patient material consists of their answers to the ORI questions, “Please give a description of yourself,” “your mother,” “father,” and posttreatment also “your therapist.” The therapists were asked: “Please give a description of your patient” and “of yourself as just that particular patient’s therapist.” The spontaneous response was followed by an “inquiry” in which the interviewer probingly repeated descriptive words used by the participant, for example, “Dutiful, what do you mean?” or “You said warm and sensitive?” The audio-recorded interviews lasted about 60 min and were transcribed verbatim. The transcripts were used for ratings of Prototype Matching of Anaclitic–Introjective Configuration and of Differentiation–Relatedness of Self and Object Representations.

### Assessment of Personality Configurations

The participants were categorized as predominantly anaclitic or introjective at baseline, following the procedure of *Prototype Matching of Anaclitic–Introjective Personality Configuration* (PMAI; Werbart and Levander 2016). The prototype-matching method (DeFife et al. 2015; Westen 2012) is close to clinical reasoning and was originally applied for diagnosing psychiatric syndromes and personality disorders. Independent assessment, rather than self-reports, has been chosen as it allows judgment of aspects of personality not directly accessible to the participant’s own experience and not confounded by the participant’s treatment experiences.

Two pairs of independent judges, blind with regard to patient identity and time in treatment, assessed the extent to which the patient respectively the therapist ORI data matched prototype descriptions of both anaclitic and introjective personality (Werbart and Forsström 2014; Werbart and Levander 2016), using a scale ranging from 1 (little or no match) to 5 (very good match). The judges were trained in PMAI ratings, using ORI interviews not included in the present study (due to missing data on some time point). Subsequently, each pair of judges performed independent ratings of half of the patient respectively the therapist data set. Cases of disagreement (a difference of two or more scale points) were discussed with the first author in order to reach a better understanding of the procedure and consensus. As a next step, the two pairs of judges rated the remaining material. Again, cases of disagreement were subject to consensus discussion. For the total patient data set, the ICC was 0.73 (presented in Werbart et al. 2017), and for the total therapist data set, the ICC was 0.68. In subsequent statistical analyses, we used the mean value of the two raters if the between-rater difference did not exceed one scale point, and consensus ratings in cases of greater disagreement. The present study combines these dimensional PMAI ratings and binary classification (categorical assessment following the highest rating on the anaclitic or the introjective dimension). For categorical classification, Cohen’s kappa for the total patient material was 0.60, and 0.63 for the total therapist material.

It has to be noted that the 66 patient PMAI ratings were based on their descriptions of themselves as a person, whereas the therapist PMAI ratings were based on their descriptions of themselves as that particular patient’s therapist. Thus, this procedure may catch the patient personality configuration merely as a “trait” and the therapist personality configuration rather as a “state.”

### Outcome Measures

Outcome measures covered both self-rated symptoms and expert-rated underlying psychological structures. *Symptom Checklist-90-R* (SCL-90; Derogatis 1994) was used to assess psychiatric symptoms experienced over the previous 7 days. The 90 items were rated on five-point Likert-scales ranging from 0 (‘not at all’) to 4 (‘very much’). For the Swedish translation of the SCL-90 a Cronbach’s alpha of 0.97 has been reported (Fridell et al. 2002). As the nine subscales are highly correlated, the Global Symptom Index (GSI) was used for further analyses.

*Differentiation–Relatedness* scale (D–R; Blatt and Auerbach 2003; Diamond et al. 1991; Huprich et al. 2016) was applied to assess developmental levels of representations of self, mother, and father. D–R assumes that, with psychological development, inner representations (i.e. cognitive–affective schemas or internal working models) of self and others become increasingly differentiated and integrated and begin to express an increased appreciation of mutual relatedness. Generally, the D–R level six or seven on the ten-point scale is regarded as the cut-off between the clinical and nonclinical range (Levy et al. 1998). A reliability study based on a part of the YAPP material reported good interrater agreement (ICC = 0.71; Hjälmdahl et al. 2001). Consensus ratings, performed by a group of trained judges, blind with regard to patient identity and time in treatment, were used in the present study.

### Data Analysis

#### Patterns of Matching Patient–Therapist Personality Configurations

The 33 therapeutic dyads were sorted into four groups according to the combination of the patient’s and the therapist’s predominant personality configuration at baseline. There are two potential convergent patterns—both assessed as anaclitic (A/A) or both assessed as introjective (I/I), and two potential complementary patterns—the patient assessed as anaclitic and the therapist as introjective (A/I), or the patient assessed as introjective and the therapist as anaclitic (I/A).

#### Outcome Patterns in the Convergent and the Complementary Groups

As a first examination of between-groups differences in outcome at termination, we calculated effect sizes for all the included outcome measures. Following the recommendations by Lakens (2013), we used Hedges’ *g* corrected for small samples (Hedges and Olkin 1985).

Subsequently, the two initially convergent and the two complementary groups were merged and compared in terms of proportion of improved and non-improved patients in terms of GSI. Patients were classified as belonging to the clinical range or functional distribution pre- and posttreatment, and at termination as improved (reliable change and crossing the cut-off between clinical and nonclinical population, or reliable change only) or as non-improved (no reliable change or reliable deterioration). Reliable change is achieved if the reliable change index, based on the difference between two time points divided by the standard error of difference, is equal to or larger than 1.96 (p < 0.05) (Jacobson and Truax 1991). For movement into a functional distribution, the cut-off between the clinical and nonclinical range was determined in accordance with Jacobson and Truax’ criterion (c), as recommended when the distributions of the functional and dysfunctional population overlap. Comparing the pretreatment YAPP sample to Swedish norms (Fridell et al. 2002), the GSI cut-off was calculated as 0.90.

Between-group differences in symptom reduction (GSI) and change in developmental levels of representations of mother, father, and self (D–R Mother, D–R Father, and D–R Self) were studied based on standardized residual gains. This procedure corrects for initial levels on outcome measures and for repeated measurements (Steketee and Chambless 1992). Treatment duration was entered as covariate in multivariate analysis of covariance (MANCOVA).

#### Power Analysis

This study was limited to 33 therapeutic dyads (16 convergent and 17 complementary). For the two-tailed t-test for independent samples with 0.05 alpha level and 0.5 effect size, the statistical power was 26%. Accordingly, the results of this preliminary investigation must be interpreted with caution.

## Results

### Patterns of Matching Patient–Therapist Personality Configurations

The categorical assessments of prototype matching resulted in 13 patients (39%) being classified as predominately anaclitic and the remaining 20 patients as predominately introjective at baseline. For the therapists, this distribution was the reverse, 20 therapists (61%) assessed as predominately anaclitic and 13 therapists as predominately introjective. Accordingly, the therapists’ mean score on the anaclitic dimension (*M* = 2.30; *SD* = 0.79) was higher than the patients’ (*M* = 2.21; *SD* = 0.86), whereas their mean score on the introjective dimension (*M* = 1.97; *SD* = 0.79) was lower than the patients’ (*M* = 2.65; *SD* = 0.85). Thus, on the group level, the therapists seem to have been more relatedness-oriented and less oriented towards self-definition than their patients. Of the 10 therapists with more than one patient six were assessed differently (as anaclitic or introjective) with different patients, and the remaining four therapists showed the same personality configuration regardless of the patient.

Of the 33 therapeutic dyads, 16 fitted in the convergent patterns of matching (8 in the A/A group and 8 in the I/I group) and 17 in the complementary patterns (5 in the A/I group and 12 in the I/A group). Mean values of the dimensional PMAI ratings in the four groups at baseline and at termination are presented in Table 1. Both patient and therapist mean PMAI ratings on the predominant dimension at baseline were at least one scale point higher than on the opposite dimension. At termination, the anaclitic patients showed better balance between relatedness and self-definition, whether belonging to the convergent or the complementary group at baseline, whereas no such improvement was observable in the introjective patients.

**Table 1:** Dimensional PMAI ratings in the four matching groups at baseline (therapists and patients) and termination (patients only)

Group	Convergent				Complementary			
	A/A		I/I		A/I		I/A	
*n*	8		8		5		12	
	M	*SD*	M	*SD*	M	*SD*	M	*SD*
Therapists								
Anaclitic	3.13	0.58	1.56	0.42	1.50	0.35	2.58	0.42
Introjective	1.44	0.42	2.88	0.52	2.70	0.27	1.42	0.36
Patients at baseline								
Anaclitic	3.13	0.64	1.56	0.50	3.00	0.00	1.71	0.50
Introjective	2.00	0.00	3.06	0.82	1.70	0.57	3.21	0.62
Patients at termination								
Anaclitic	2.56	0.62	1.75	0.76	2.20	0.84	1.92	0.87
Introjective	2.19	0.75	2.88	0.58	2.30	0.84	2.88	0.48

A/A = both assessed as anaclitic; I/I = both assessed as introjective; A/I = anaclitic patient with introjective therapist; I/A = introjective patient with anaclitic therapist

### Outcome Patterns in the Convergent and the Complementary Group

Descriptive statistics of outcome data and effect sizes for the four groups of matching patterns are presented in Table 2. On the GSI at termination, both convergent groups (A/A and I/I) crossed the cut-off between the clinical and the nonclinical population (0.90), whereas the means for the two complementary groups (A/I and I/A) remained on the clinical level. On the D–R scale, the means for all the four groups were in the nonclinical range at termination. In terms of symptom reduction, the two convergent groups improved with large effect sizes at termination (Hedges’ *g* > 0.8), whereas the effect sizes in the two complementary groups were small (0.2 < Hedges’ *g* < 0.5). The developmental level of representations of self and parents improved with large effect sizes for the anaclitic patients in both the convergent and the complementary group, whereas the introjective patients in both groups had small to medium effect sizes on the three D–R measures.

**Table 2:** Descriptive statistics of outcome data and effect sizes for four groups of matching patterns

Outcome measure	A/A group				I/I group				
	*n*	M	*SD*	Hedges’ *g* (corrected)	*n*	M	*SD*	Hedges’ *g* (corrected)	*N* total
GSI									
T1	8	1.75	0.78		8	1.40	0.51		16
T2	7	0.69	0.62	1.39	7	0.56	0.25	1.92	14
D–R Mother									
T1	8	6.50	0.93		8	7.13	0.83		16
T2	8	7.75	0.71	1.43	8	7.13	0.99	0.00	16
D–R Father									
T1	8	6.50	0.93		8	6.63	1.51		16
T2	8	7.38	0.74	0.99	8	7.38	1.06	0.54	16
D–R Self									
T1	8	6.25	1.04		8	6.75	1.04		16
T2	8	7.38	0.74	1.18	8	7.00	2.20	0.14	16

Outcome measure	A/I group				I/A group				
	*n*	M	*SD*	Hedges’ *g* (corrected)	*n*	M	*SD*	Hedges’ *g* (corrected)	*N* total
GSI									
T1	5	1.38	0.59		12	1.24	0.64		17
T2	5	1.17	0.39	0.38	11	0.98	1.09	0.29	16
D–R Mother									
T1	5	5.00	2.24		12	6.67	1.23		17
T2	5	7.00	1.22	1.00	12	6.67	1.07	0.00	17
D–R Father									
T1	5	5.40	2.07		12	6.67	1.07		17
T2	5	7.00	1.22	0.85	12	7.25	0.87	0.58	17
D–R Self									
T1	5	4.60	2.41		12	6.25	1.29		17
T2	5	6.80	1.79	0.94	12	7.17	1.11	0.73	17

*n* varies due to the varying number of respondents at each assessment. T1 = pre-treatment; T2 = termination

In order to increase sample sizes for further statistical analyses, we merged the four groups into two overarching patterns: the convergent and the complementary matching group. Pretreatment, the between-group differences in symptom severity and levels of Differentiation–Relatedness were not significant. A one-way MANCOVA (based on standardized residual gain scores) revealed a significant between-group difference in outcome for the four measures taken together [*F* (4, 24) = 2.94, *p* = 0.041; Wilks’ Lambda = 0.67, partial *η*^2^ = 0,329] with larger improvement in the convergent group. A post-hoc analysis of each outcome measure, using a Bonferroni adjusted alpha level, showed significantly larger improvements in the convergent group for GSI [*F* (1, 27) = 6.22, *p* = 0.019, partial *η*^2^ = 0.187] and for D–R Mother [*F* (1, 27) = 5.81, *p* = 0.023, partial *η*^2^ = 0.177]. No significant between-group differences were found for the D–R Father and D–R Self.

In terms of individual patterns of change in symptom severity (GSI), 28 of the 33 patients (85%) belonged to the clinical range pretreatment (Table 3). At termination, 17 of the 30 patients with outcome data (57%) showed reliable improvements (both patients with clinically significant symptom reduction and patients with reliable change only). The proportion of improved patients was twice as large in the convergent group (79%) than in the complementary group (38%).

**Table 3:** Patients below and above clinical cut-off, improved patients (clinically significant improvement or reliable change only) and non-improved patients (no reliable change or deterioration) in the convergent and the complementary group

GSI	Pre-treatment			Termination		
	Convergent	Complementary	Total	Convergent	Complementary	Total
*n*	16 (%)	17 (%)	33 (%)	14 (%)	16 (%)	30 (%)
Clinical range	15 (94)	13 (76)	28 (85)	1 (7)	7 (44)	8 (27)
Functional distribution	1 (6)	4 (24)	5 (15)	13 (93)	9 (56)	22 (73)
Improved	–	–	–	11 (79)	6 (38)	17 (57)
Non-improved	–	–	–	3 (21)	10 (63)	13 (43)
Missing data				2	1	3

*n* varies due to the varying number of respondents at each assessment. Cut-off between clinical and nonclinical population for GSI = 0.90

## Discussion

The present study is, according to our knowledge, the first attempt to explore matching of patient and therapist personality configuration in terms of Blatt’s (2008) two-polarities model in relation to outcome. Most therapists were assessed as predominantly anaclitic, whereas most patients were assessed as introjective. Half of the therapeutic dyads fitted in the convergent pattern of matching and the other half in the complementary pattern. Analysis of between-group differences showed large effect sizes, in terms of symptom reduction at termination, in the two convergent groups (both oriented on relatedness or both oriented on self-definition) and small effect sizes in the two complementary groups (dyads with opposite orientations). More than twice as many patients in the convergent dyads showed reliable change at termination, as compared to the complementary dyads. Changes in the developmental level of representations of self and others displayed another pattern, with large effect sizes for anaclitic patients and small to medium effect sizes for introjective patients. Thus, these results indicate that the patient–therapist personality match at pre-treatment does matter for symptom reduction at termination, whereas changes in the developmental levels of representations of self and others seem to be dependent on the patient’s predominant personality configuration pre-treatment. This is consistent with the double helix model, claiming direct link between the polarities of self-definition–relatedness and the dimensions of differentiation–relatedness in representations of self and others, both in personality development and in the therapeutic process (Blatt et al. 2008; Luyten and Blatt 2013).

Accordingly, a previous investigation of changes in the anaclitic–introjective personality configuration following psychotherapy in the same patient group (Werbart et al. 2017) showed better balance between relatedness and self-definition post-treatment in the initially anaclitic patients, whereas this improvement was not significant in the initially introjective patients. However, no significant between-group differences could be found on outcome measures. The present study demonstrated better outcomes in terms of GSI and D–R Mother in the merged convergent group than in the merged complementary group. A comparison of these two preliminary studies suggests that the lack of differences in outcome between the anaclitic and the introjective patients might conceal consequences of the patient–therapist personality match at the outset of psychotherapy. Consequently, our study confirms that regarding the patient–therapist dyad as a distinct variable, as suggested by Silberschatz (2017), may result in new and clinically highly relevant findings, not attainable when regarding the patient and the therapist variables separately.

Still, our study suggests a potential within-therapist effect, besides the effects of patient–therapist match. Six of the 10 therapists with more than one patient were assessed as having different personality configurations with different patients. These therapists could be more relatedness-oriented with anaclitic patients and more self-definition oriented with introjective patients. Thus, different personality configurations could be actualized in the same therapist early in the relationship with different patients. This implies that the therapists’ personality configuration, as it manifests itself in relationship to their patients, might differ from their configuration in private relationships (cf. Heinonen and Orlinsky 2013). Zilcha-Mano (2017) differentiated between more stable, “trait-like,” and more interaction-related, “state-like,” tendencies to form satisfying relationships with others, the latter making alliance therapeutic. Our study demonstrates, accordingly, that the therapist’s “state-like” interpersonal stance plays a role. The therapists’ capacity to adjust their balance between relatedness and self-definition early in the therapeutic process to the patients’ “trait-like” personality configurations was connected with better outcomes. Furthermore, it is possible that the therapists who did not alter their interpersonal stance despite their patients’ personality configurations are those for whom their “trait-like” personality configuration had pervasive influence on their ways of being with patients, potentially leading to less optimal outcomes.

Contrary to our expectations, the present study showed more pronounced improvements in the convergent group of patient–therapist matching personality configuration, thus supporting the non-complementarity or similarity hypothesis (Taber et al. 2011; Wiseman and Tishby 2014) and contradicting the complementarity hypothesis (Bruck et al. 2006; Kiesler and Watkins 1989; Petrowski et al. 2011). However, our study does not answer the question why convergent personality configuration in therapeutic dyads is connected with more pronounced improvements. We can only speculate that matching interpersonal stance facilitates early “moments of meeting” (Stern et al. 1998), thus promoting working alliance and fruitful therapeutic work. Furthermore, our study is limited to the initial match. Hypothetically, the patient–therapist matching is not a static variable but an ongoing process. Our finding that some therapists can better than others adjust their interpersonal stance to the patient’s personality configuration, is consistent with Mallinckrodt’s (2010) suggestion that effective therapists can offer their patients progressively changing series of relationships, creating a corrective emotional experience that promotes more adaptive functioning. Accordingly, a study of anaclitic and introjective patients in psychoanalysis suggested that the psychoanalytic technique has to be adjusted to the anaclitic and introjective patients’ different needs and defenses, in order to reactivate developmental processes (Werbart and Levander 2016). Thus, the complementarity hypothesis might be still valid later on in the therapeutic process, in the middle phase of working through, and has to be tested in further studies.

### Strengths, Limitations and Future Directions

The present study contributes to our knowledge of the role of the patient’s and the therapist’s personality configuration in creating productive therapeutic dyads. Rather than studying match on directly observable, explicit characteristics (such as gender, socio-economic status, ethnicity, etc.) or the participants’ own subjective experiences of match, we focus on implicit, “deep” personality features, highly relevant for psychotherapy process, and in consequence, for treatment outcomes. Furthermore, no previous study investigated the therapists’ personality configuration (orientation on relatedness or self-definition) as actualized early in the therapeutic process. The need of studies focusing on effects of the patient–therapist match as a single variable has been repetitively stressed in psychotherapy research (cf. Taber et al. 2011; Zilcha-Mano 2017). Our approach, regarding the patient–therapist dyad as a distinct variable (Silberschatz 2017), made it possible to discover differences in outcomes between the convergent and the complementary therapeutic dyads, not visible in a previous comparison of outcomes for anaclitic and introjective patients (Werbart et al. 2017).

The main limitation of this study is its small sample size. The low statistical power limits the possibility of finding genuine between-group differences, at the same time as the positive findings are less likely to be true positive. Thus, this research should be conducted at larger scale. As a consequence of the naturalistic design, we cannot claim that the observed between-group differences were real effects of patient–therapist matching. Furthermore, the sample is not representative of young adult outpatients in other forms of long-term treatment or of more homogenous diagnostic groups. The treatment duration varied greatly and could potentially affect the outcome. In our statistical analysis, we entered duration as a covariate, thus controlling for its effects. Studying young adults in psychotherapy raises the question of maturational processes in this dynamic period of life and of spontaneous improvement. However, we may assume that these effects should be equal in the two groups.

Future studies, with larger number of therapists treating several patients, can address the between-therapists differences in adjusting their relational stance to their patients’ personality configurations. Lacking recordings of therapy sessions we could not study potential changes in the therapists’ adjustment (or maladjustment) to their patients’ changing dynamics between relatedness and self-definition during the course of treatment. The interviews with patients and therapists varied in quality and were not especially designed for PMAI ratings. Session recordings could be used for PMAI expert ratings, together with self-rating measures of the two dimensions of personality configurations, for example applying the Dysfunctional Attitude Scale or the Big Five Personality Test.

### Clinical Implications

One implication of our study is the importance of early understanding of the patient’s predominant interpersonal stance in terms of focus on relatedness/emotional bond or self-definition/autonomy. Furthermore, it is the therapist’s task to observe which aspects of the therapist’s “trait-like” interpersonal stance are actualized and staged early in the therapeutic relationship. Therapists might expect to feel and react differently depending on whether their characteristic focus on relatedness or self-definition is convergent or complementary to the patient’s personality configuration. Blatts’ two-polarities model enables therapists to reflect on the dynamics in the therapeutic relationship, starting from the interplay between the patient’s and the therapist’s personality configuration. Being aware of both participants’ relational stance, the therapist might form an idea of the patient’s capability to establish an attachment to the therapist, the patient’s potential reactions to therapeutic boundaries and separations, and to different kinds of interventions. Based on this knowledge, it can be possible for the therapist to adjust to the patient’s way of relating and safeguarding own self-boundaries, thus potentially facilitating the change process and enhancing the outcome. The therapists might be more able to work through ruptures in the working alliance and potentially prevent negative outcomes by monitoring, from the beginning, the patient’s ways of being with the therapist, as well as their own ways of being with the patient. Measures of the patient’s and the therapist’s personality configurations can be included in systems for monitoring patient progress and for feedback-informed treatments (Lambert et al. 2005; Miller et al. 2015). The interplay between the patient’s and the therapist’s personality configurations together co-creates the unique situation within which change is possible. Our study suggests that this should be considered already in the beginning of therapy.
