# Correction to: Matching Patient and Therapist Anaclitic–Introjective Personality Configurations Matters for Psychotherapy Outcomes

**DOI:** 10.1007/s10879-018-9391-1

**Published:** 2018-04-03

**Authors:** Andrzej Werbart, Mikael Hägertz, Nadja Borg Ölander

**Affiliations:** Department of Psychology, Stockholm University, 106 91 Stockholm, Sweden

## Correction to: Journal of Contemporary Psychotherapy 10.1007/s10879-018-9389-8

The original version of this article unfortunately contained a mistake in the co-author name. The co-author name should be Nadja Borg Ölander instead it was published as Nadja Berg Ölander.

The original article has been corrected.

